# Immune-checkpoint inhibitor-induced diarrhea and colitis in patients with advanced malignancies: retrospective review at MD Anderson

**DOI:** 10.1186/s40425-018-0346-6

**Published:** 2018-05-11

**Authors:** Yinghong Wang, Hamzah Abu-Sbeih, Emily Mao, Noman Ali, Faisal Shaukat Ali, Wei Qiao, Phillip Lum, Gottumukkala Raju, Gladis Shuttlesworth, John Stroehlein, Adi Diab

**Affiliations:** 1 Department of Gastroenterology, Hepatology and Nutrition, The University of Texas MD Anderson Cancer Center, 1515 Holcombe Blvd, Houston, TX 77030 USA; 2 Department of Internal Medicine, Baylor College of Medicine, Houston, TX USA; 3 Department of Biostatistics, The University of Texas MD Anderson Cancer Center, Houston, TX USA; 4 Department of Melanoma Medical Oncology, The University of Texas MD Anderson Cancer Center, Houston, TX USA

**Keywords:** Immune checkpoint inhibitor, Diarrhea, Colitis, Overall survival

## Abstract

**Background:**

Immune checkpoint inhibitors (ICPIs) are gaining increasing popularity as an efficacious treatment for advanced malignancies. ICPI treatment can be complicated by diarrhea and colitis. Systemic steroids are the first line treatment. Infliximab is reserved for severe refractory cases. We aimed to assess the impact of ICPI-induced diarrhea and colitis and their immunosuppressive treatment on patients’ outcomes.

**Methods:**

This retrospective analysis was conducted in 327 cancer patients who received ICPIs between 2011 and 2017. Patients with ICPI-induced toxicities in other organs were excluded. We collected data about patient demographics, clinical variables, and overall survival. We used descriptive analysis to compare different groups based on the occurrence and the treatment of diarrhea and colitis. Kaplan-Meier and log-rank test were used to estimate and compare overall survival durations between groups.

**Results:**

Diarrhea was recorded in 117 (36%) patients; 79 (24%) of them required immunosuppressive treatment of either systemic corticosteroid without infliximab (*n* = 44) or with infliximab (*n* = 35). Caucasian ethnicity, melanoma, stage 3 cancer, and ipilimumab were predictors of colitis that requires immunosuppression. Patients who required immunosuppressants had better overall survival than those who did not require treatment for colitis or diarrhea (*P* < 0.001). Immunosuppression for diarrhea or colitis did not affect the overall survival significantly (*P* = 0.232), nor did the choice of treatment (corticosteroids with vs. without infliximab; *P* = 0.768). Diarrhea was an independent predictor of a favorable overall survival (*P* < 0.001), irrespective of treatment need (*P* = 0.003). We confirmed the same results in a subgroup analysis for patients with stage IV malignancies only. Patients who received long duration of steroid treatment (> 30 days) had numerically higher infection rate than those who received steroid for shorter duration (40.4 vs. 25.8%, *P* = 0.160). Likewise, long duration of steroid without infliximab was associated with increased risk of infection compared to short duration of steroid with infliximab (42.9% vs. 14.3%, *P* = 0.089).

**Conclusions:**

Patients with ICPI-induced diarrhea or colitis have improved survival outcomes. Diarrhea is an independent predictor of an improved survival regardless of treatment requirement. Immunosuppressive treatment for diarrhea did not significantly affect overall survival, however, infection rates were numerically higher among patients who received steroids for a long duration. Therefore, early non-steroid immunosuppressive therapy may ensure a more favorable overall outcome.

## Background

Oncogenesis is a multifactorial phenomenon in which suppression of host immune response against the tumor is of prime importance [1]. Cancer therapy has monumentally changed in recent years with the advent of immune checkpoint inhibitors (ICPIs), agents that are primed to enhance immunity against tumor cells by blocking ligands or proteins that would otherwise lead to inactivation or death of cytotoxic antitumor T-cells. Programmed death-1/ligand 1 (PD1/L1) inhibitors, and cytotoxic T-lymphocyte-associated protein-4 (CTLA-4) inhibitors have shown significant potential in improving overall survival (OS) in patients with malignant melanoma, non-small cell lung carcinoma, and renal cell carcinoma [2–6]. Multiple ICPI clinical trials have shown favorable response rates in various forms of malignancies, and the indications for the use of ICPIs are anticipated to grow substantially in the future [4, 7–9]. With this foresight, it is important to have a thorough understanding of the toxicity profile of these agents, which, although favorable compared with traditional chemotherapy, can be dose-limiting and fatal in severe cases.

Because ICPIs lead to generalized activation of T-cells, the non-specific infiltration of these immune cells can affect virtually any organ system of the body. Commonly observed toxicities, often referred to as immune-related adverse events (irAEs), affect the endocrine system, gastrointestinal (GI) tract, liver, and lungs**.** GI tract irAEs typically present with diarrhea, which could be either the only presenting symptom that is self-limiting or part of ICPI-induced colitis that requires hospitalization and treatment. Most clinical trials have reported GI tract toxicities as the most commonly recorded serious irAEs [10, 11]. Additionally, ICPI induced GI toxicities are the most common reason for immunotherapy treatment discontinuation [11]. Regimens containing CTLA-4 agents are more likely to cause GI toxicities [12]. On the other hand, PD-1 or PD-L-1 monotherapies are associated with lower risk of GI toxicities [13, 14].

Because irAEs are autoimmune entities, they should be reversed by immunosuppression, with corticosteroids as the first-line agent in the current practice. Infliximab is usually reserved for the treatment of GI-irAEs that are refractory to steroids or of high severity. However, adverse events are proposed to be indicators of good tumor response [15, 16]. Hence, steroid-induced immunosuppression may potentially hamper the antitumor effect of ICPIs. The impact of steroids on tumor response to ICPI treatment is not very well understood and the medical literature is sparse on this particular issue.

The aim of our study, in which we describe our experience as a major cancer center, was to investigate the impact of ICPI-induced diarrhea and colitis and their treatment with corticosteroids and infliximab on patients’ overall survival.

## Methods

### Study design and population

We conducted a retrospective, descriptive, single-center study after obtaining approval from the Institutional Review Board at The University of Texas MD Anderson Cancer Center. We investigated adult cancer patients who received ICPIs at MD Anderson between March 2011 and March 2017. Adult cancer patients who received PD-L1 inhibitors, PD-1 inhibitors, or CTLA-4 inhibitors, as single agents or as multiple-agent therapy for a malignancy under a clinical trial or otherwise, were included. Patient selection was not governed by whether the ICPIs were administered after failure of multiple other cancer treatment regimens or as a first-line treatment. Patients who developed and/or were treated for ICPI-induced adverse events other than diarrhea or colitis (e.g., endocrine, dermatologic, or pulmonary irAEs) were excluded. Patients who had diarrhea or colitis and other concomitant irAEs were excluded in our analysis. We extracted patient data from institutional electronic medical charts and pharmacy databases. Patients who had positive GI infectious disease at the onset of symptoms were excluded from our analysis.

Once our study cohort was identified, we collected data regarding patient demographics, comorbidities, medical and oncologic history, cancer treatment regimen(s), ICPI regimen(s), irAEs and related variables, management of irAEs and their treatment complications, and OS. Pertinent variables collected from medical history included 1) general demographic information; 2) comorbidities documented in patients’ charts, including coronary artery disease, congestive heart failure, chronic obstructive pulmonary disease, human immunodeficiency virus, atrial fibrillation, graft-versus-host disease, asthma, hypertension, diabetes mellitus, dyslipidemia, and hypocorticolism; 3) underlying autoimmune diseases, including celiac disease, rheumatoid arthritis, psoriasis, systemic lupus erythematosus, connective tissue disease, Hashimoto thyroiditis, sarcoidosis, and Sjogren’s syndrome; 4) smoking history; 5) current or prior (within 3 months of ICPI initiation) use of nonsteroidal anti-inflammatory agents; and 6) performance status scores documented in patients’ charts at the time of ICPI initiation according to the Eastern Cooperative Oncology Group (ECOG) scoring system [17].

Oncologic history was screened for variables relating to the following: 1) cancer type, 2) cancer stage based on the American Joint Committee on Cancer staging system, and 3) ICPI agents. We categorized malignancies as hematologic, melanoma, or solid tumor malignancies. Non-melanoma skin malignancies were included in our cohort. We did not include cancer staging for hematological malignancies. CPI agents were mostly listed individually; combinations were defined as CTLA-4 and PD-1 dual regimens. Regarding irAEs, the incidence, irAE type, medications employed for treatment of irAEs, and duration of irAE treatment were recorded. Duration of steroid use was defined as the initiation of high doses of steroids at the time of irAE diagnosis to the time of cessation or back to previous baseline maintenance dose as part of the cancer therapy regimen. We arbitrarily used 30 days as the cutoff to indicate short duration (≤30 days) and long duration (> 30 days) of steroid use. Additional data related to GI-irAEs recorded included 1) diarrhea or colitis grade at the time of diagnosis of the irAE based on the National Cancer Institute’s Common Terminology Criteria for Adverse Events, version 4.03., 2) infection following steroid initiation up to 1 month after steroid treatment completion, and 3) overall survival duration.

### Outcomes after GI-irAEs

Infection as a complication from immunosuppressive treatment was recorded. Infections were diagnosed on the basis of evaluation of diagnostic laboratory tests (including blood, stool, urine, and sputum culture), imaging, fluid aspiration, or other diagnostic test. Infections diagnosed following initiation of immunosuppressive agents up to 1 month after the end of the immunosuppressive treatment were considered a complication of the irAE treatment. OS was defined as the time from initial exposure to ICPIs until the time of death or last follow-up, whichever occurred first.

### Statistical analysis

Statistical analysis was carried out using SAS version 9.4 (SAS Institute, Cary, NC) and TIBCO Spotfire S+ version 8.2 (TIBCO Software Inc., Palo Alto, CA). The distribution of each continuous variable was summarized using means, standard deviations, and ranges. The distribution of each categorical variable was summarized using frequencies and percentages. Continuous variables were compared among groups using the Kruskal-Wallis test for more than two groups. The Fisher exact test or chi-square test was used to evaluate associations between two categorical variables. Kaplan-Meier curves were used to estimate unadjusted OS. Log-rank test was used to compare OS between groups. All statistical evaluations were two-sided. *P* values of less than 0.05 were considered statistically significant.

## Results

### Clinical features

Following the exclusion of patients who had ICPI-induced adverse events involving other organs, a total of 327 patients were included in our analysis. Diarrhea was observed in 117 (36%) patients. Seventy-nine (24%) patients needed immunosuppressive treatment with either systemic corticosteroids without infliximab (*n* = 44) or with infliximab (*n* = 35). Thirty-eight (12%) patients had diarrhea but did not receive treatment with steroids or infliximab. Patient demographics are shown in Table 1. Age, sex, comorbidities, underlying autoimmune disorders, smoking status, and history of nonsteroidal anti-inflammatory agent use were similar among different groups.

**Table 1:** Patient characteristics

	No. (%)			
Characteristic	Diarrhea treated with immunosuppressants, *n* = 79	Diarrhea without treatment, *n* = 38	No diarrhea, *n* = 210	*P*
Mean age (years, SD)	59.8 (15)	62.2 (12)	59.2 (14)	0.480
Male sex	53 (67)	25 (66)	133 (63)	0.825
Race				< 0.001
White	75 (95)	32 (84)	154 (73)	
Other	4 (5)	6 (16)	56 (27)	
Comorbidities present	24 (30)	9 (24)	62 (30)	0.733
Underlying autoimmune disorder	4 (5)	1 (3)	13 (6)	0.663
History of smoking	36 (46)	20 (53)	107 (51)	0.671
History of use of nonsteroidal anti-inflammatory agents	38 (48)	15 (39)	80 (38)	0.300
ECOG performance status				0.070
0–2	78 (99)	38 (100)	197 (94)	
3–4	1 (1)	0 (0)	13 (6)	
Malignancy type				< 0.001
Melanoma	55 (70)	9 (24)	56 (27)	
Solid tumor	23 (29)	14 (37)	111 (53)	
Hematologic	1 (1)	15 (39)	43 (20)	
Cancer Stage^a^				< 0.001
Stage III	16 (21)	2 (9)	7 (4)	
Stage IV	61 (79)	21 (91)	149 (96)	
Colitis grade 2–3	49 (62)	–	–	–
Checkpoint inhibitor type				< 0.001
Ipilimumab	48 (61)	23 (61)	67 (32)	
Nivolumab	5 (6)	8 (21)	87 (41)	
Pembrolizumab	13 (16)	6 (16)	50 (24)	
Combination^b^	12 (15)	1 (3)	6 (3)	
Atezolizumab	1 (1)	0 (0)	0 (0)	

Abbreviations: ECOG, Eastern Cooperative Oncology Group; SD, standard deviation

^a^The American Joint Committee on Cancer staging system was used. Stage was known in 77 patients in the immunosuppressant group, 23 in the untreated diarrhea group, and 156 in the no diarrhea group

^b^Combination therapy consisted of ipilimumab + nivolumab

The group of patients who needed immunosuppressive treatment included more Caucasian patients than did other groups (95% vs. 73 and 84%; *P* < 0.001). The immunosuppressant group had a significantly higher proportion of patients with melanoma than other groups (70% vs. 24 and 27%; *P* < 0.001). Likewise, patients with stage III malignancies were found in the immunosuppressant group more than other groups (21% vs. 4 and 9%; *P* < 0.001). A significantly higher proportion of patients in the immunosuppressant group received ipilimumab either alone or in combination with nivolumab compared to the other groups (76% vs. 35 and 64%; *P* < 0.001). Table 2 summarizes data related to different ICPI agents stratified by malignancy type. The ICPI treatment regimen in the melanoma group consisted of ipilimumab as a single agent or combination therapy more often than other malignancy groups (76% vs. 22 and 37%; *P* < 0.001).

**Table 2:** Immune checkpoint inhibitor agent administered by malignancy type

	No. (%)			
Checkpoint inhibitor	Melanoma *n* = 120	Solid tumor *n* = 148	Hematologic cancer *n* = 59	*P*
Ipilimumab	*81 (68)*	44 (30)	13 (22)	< 0.001
Nivolumab	*7 (6)*	62 (42)	31 (53)	
Pembrolizumab	*23 (19)*	31 (21)	15 (25)	
Combination^a^	*9 (8)*	10 (7)	0 (0)	
Atezolizumab	*0 (0)*	1 (1)	0 (0)	

^a^Combination therapy consisted of ipilimumab + nivolumab

Stage IV malignancies were associated with a lower incidence of diarrhea and colitis when compared to patients with stage III malignancies (35.3% vs. 72.0%; *P* = 0.001), and less infliximab treatment (9.9% vs. 48%; *P* < 0.001). The percentage of patients received steroid treatment without infliximab and the duration of steroid treatment were similar between the two groups (Table 3).

**Table 3:** Association of cancer stage and irAE in patients with melanoma or solid tumor

	Cancer stage III *n* = 25	Cancer stage IV *n* = 232	*P*
Diarrhea/colitis^a^	*18 (72.0)*	82 (35.3)	0.001
IrAE immunosuppressant treatment^b^			
Steroid alone	*4 (16.0)*	38 (16.4)	1.000
Steroid/Infliximab	*12 (48.0)*	23 (9.9)	< 0.001
Mean length of steroid treatment (days, SD)	*59 (33)*	59 (85)	0.962

*Abbreviations*: *irAE*, ICPI related adverse event; *SD*, standard deviation

^a^Total 17 patients with diarrhea/colitis had missing staging information

^b^Immunosuppressant treatment was administrated in total 77 patients

Patients with higher odds of developing diarrhea or colitis were Caucasian patients (OR, 5.76; 95% CI, 2.03–16.36; *P* = 0.001), diagnosed with melanoma (OR, 1.96; 95% CI, 1.04–3.67; *P* = 0.037), and/or received ICPI regimens containing ipilimumab (OR, 2.23; 95% CI 1.03–4.81; *P* = 0.041). In contrast, patients with stage IV cancer were found to have lower odds of developing diarrhea or colitis (OR, 0.09; 95% CI, 0.03–0.3; *P* < 0.001; Table 4). Additionally, shorter duration of ICPI treatment was associated with more irAEs (OR, 0.99; 95% CI, 0.99–1.00; *P* = 0.049).

**Table 4:** Multivariate logistic regression for colitis risk

	IrAE^a^	
Characteristic	OR (95% CI)	*P*
Race (Caucasian)	5.76 (2.03–16.36)	0.001
Cancer stage IV	0.09 (0.03–0.30)	< 0.001
Cancer type (Melanoma)	1.96 (1.04–3.67)	0.037
ICPI agent		
Ipilimumab + combination	2.23 (1.03–4.81)	0.041
Nivolumab	0.39 (0.14–1.11)	0.079
Duration of ICPI treatment	0.99 (0.99–1.00)	0.049

^a^Race, cancer stage, cancer type, ICPI agent, and duration of ICPI treatment were included in the multivariate logistic regression for colitis risk

### Steroids alone compared to steroids with infliximab

Table 5 shows clinical characteristics of the subgroup of patients who developed diarrhea requiring immunosuppressive treatment. Diarrhea of grade 2 and higher was associated with an increased need for the addition of infliximab to corticosteroids treatment (97% vs. 73%; *P* = 0.005). The immunosuppressive treatment was not different according to the grade of colitis (*P* = 0.163). The mean time to diarrhea onset was similar between the groups (*P* = 0.976).

**Table 5:** Associations between diarrhea or colitis treatment and severity

	No. (%)		
Clinical characteristics	Steroids only *n* = 44	Steroids/infliximab *n* = 35	*P*
Colitis grade			0.163
1	*20 (45)*	10 (29)	
2–3	*24 (55)*	25 (71)	
Diarrhea grade			0.005
1	*12 (27)*	1 (3)	
2–3	*32 (73)*	34 (97)	
Mean time in days to diarrhea onset (SD)	*80 (130)*	81 (96)	0.976

### Endoscopic and histological evaluation

In our study, 53 (67.1%) of the patients who had diarrhea and received immunosuppressive treatment had endoscopic evaluation. Endoscopically, 43 patients had active inflammation and 10 had normal endoscopic appearance. Histologically, 48 patients had features suggestive of ICPI-induced colitis and 5 had normal histology. Endoscopic and histological characterization of our cohort had been described in details in our separate study that was accepted by IBD journal “Endoscopic and Histologic Features of Immune Checkpoint Inhibitor-Related Colitis” [18].

### Impact of ICPI-induced diarrhea and immunosuppressive treatment on outcomes

**Fig. 1:**
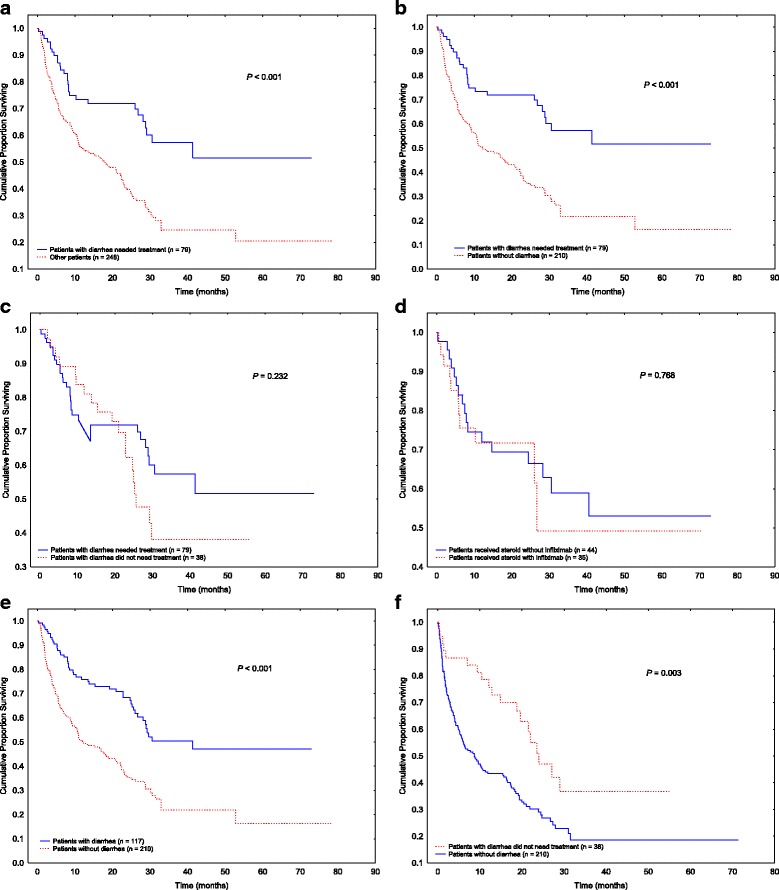
Overall survival in various patient groups. **a** Patients with diarrhea or colitis requiring treatment compared with patients who had no or mild symptoms not requiring treatment. **b** Patients with diarrhea or colitis requiring treatment compared with patients who did not develop diarrhea. **c** Patients with diarrhea requiring treatment compared with those with diarrhea not requiring treatment. **d** Patients with diarrhea treated with steroids alone compared with those requiring infliximab. **e** Patients who developed diarrhea compared with those who did not. **f** Patients with diarrhea that did not require treatment compared with those who did not develop diarrhea

Analysis of OS showed that patients with ICPI-induced diarrhea or colitis that required treatment had significantly better OS duration than those without diarrhea that required treatment owing to either lack of or mild symptoms (Fig. 1a; *P* < 0.001). Significant differences were observed between patients with diarrhea or colitis requiring treatment and those who did not have diarrhea (Fig. 1b; *P* < 0.001). Among patients who developed diarrhea or colitis, the use of immunosuppressive treatment did not appear to impact OS significantly (Fig. 1c; *P* = 0.232). OS also did not differ between patients treated with steroids alone and those requiring infliximab for steroid-refractory diarrhea or colitis (Fig. 1d; *P* = 0.768). Overall, patients who developed diarrhea had better OS duration than those who did not develop diarrhea (Fig. 1e; *P* < 0.001). The difference in OS duration persisted and remained statistically significant between patients with mild diarrhea who did not need any immunosuppressive treatment and patients without diarrhea (Fig. 1f; *P* = 0.003).

### Impact of malignancy stage on outcome

**Fig. 2:**
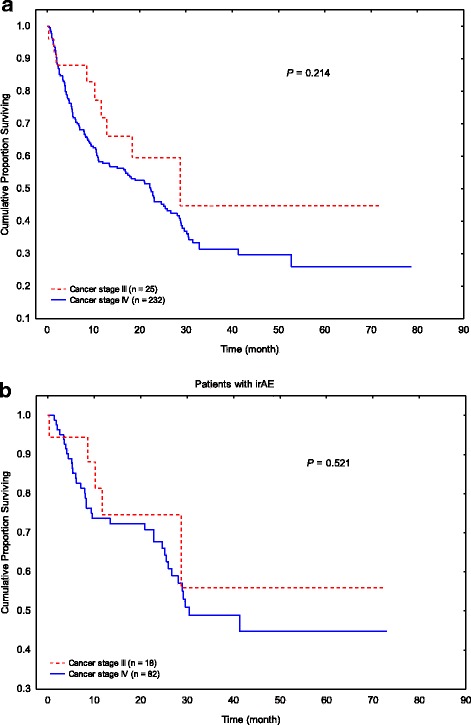
Overall survival in patients with different cancer stages. **a** All patients with stage III melanoma and solid tumor malignancies compared with stage IV. **b** GI irAE patients with stage III melanoma and solid tumor malignancies compared with stage IV

OS duration did not differ by the stage of malignancy in patients with melanoma or solid tumor (stage III, 25 patients vs. stage IV, 232 patients; Fig. 2a; *P* = 0.214). Even in the subgroup of patients with GI tract irAE, the OS duration remained comparable between the two groups (Fig. 2b; *P* = 0.521).

**Fig. 3:**
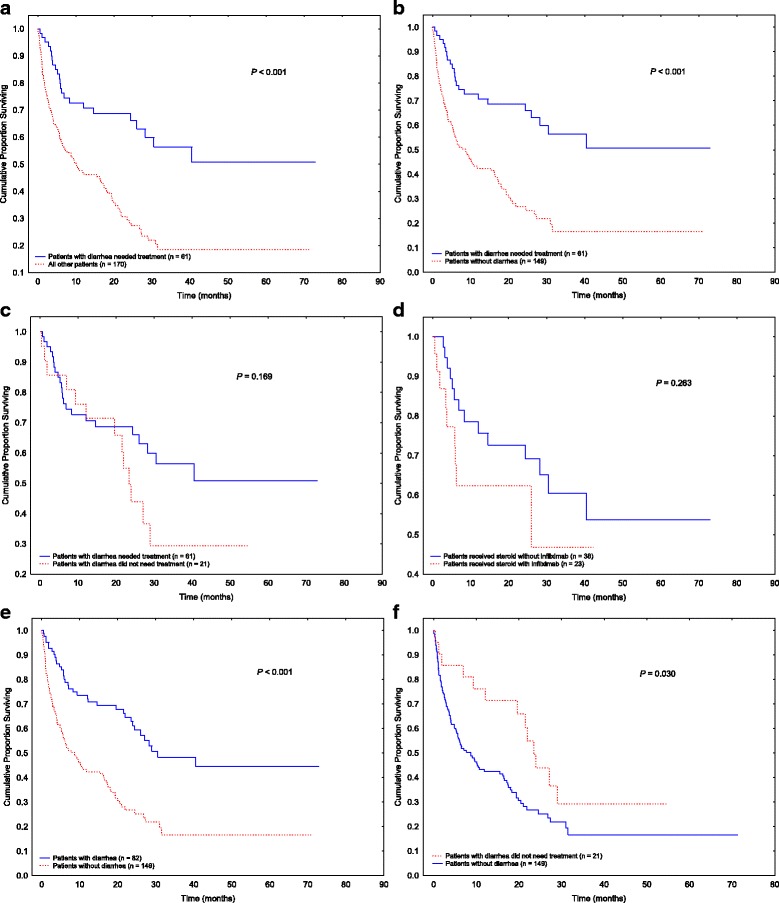
Overall survival in patients with stage IV disease. **a** Patients with diarrhea or colitis requiring treatment compared with patients who had no or mild symptoms not requiring treatment. **b** Patients with diarrhea or colitis requiring treatment compared with patients who did not develop diarrhea. **c** Patients with diarrhea requiring treatment compared with those with diarrhea not requiring treatment. **d** Patients with diarrhea treated with steroids alone compared with those requiring infliximab. **e** Patients who developed diarrhea compared with those who did not. **f** Patients with diarrhea that did not require treatment compared with those who did not develop diarrhea

Among patients with stage IV cancer, we observed a similar pattern as in the entire cohort (OS curves demonstrated in Fig. 1); patients who needed treatment for diarrhea or colitis had better OS duration than those who did not need immunosuppressive treatment (Fig. 3a; *P* < 0.001). Significant differences were observed between patients with diarrhea or colitis requiring treatment and those without diarrhea (Fig. 3b; *P* < 0.001). Among patients who developed diarrhea or colitis, the use of immunosuppressive treatment did not impact OS significantly (Fig. 3c; *P* = 0.169). OS did not differ between patients treated with steroids alone and those who needed infliximab for steroid-refractory diarrhea or colitis (Fig. 3d; *P* = 0.263). Also among only patients with stage IV cancer, those who developed diarrhea had better OS duration than did those without diarrhea (Fig. 3e; *P* < 0.001), and this difference persisted even in patients with diarrhea that did not require treatment (Fig. 3f; *P* = 0.030).

### Duration of steroid use and outcomes

The duration of steroid treatment was categorized as short duration in 30 (38%) patients and long duration in 49 (72%) patients. In patients who received a short duration of steroid use, death was the cause of short treatment duration in only 2 patients with the rest all achieved clinical response. We found infections in 29 (37%) patients among the 79 patients who had diarrhea or colitis and received immunosuppressive treatment (Table 6). The mean duration of steroid use in patients who developed an infection was longer than in those who did not develop an infection (80.4 days vs. 46.7 days; *P* = 0.063). The median length of steroid treatment in patients with an infection was 60 days (interquartile range [IQR], 7–650 days), and in patients without an infection was 34 days (IQR, 8–161 days). Otherwise, no significant differences were observed between patients who developed an infection and those who did not in terms of cancer stage, malignancy type, ICPI agent, diarrhea grade, or colitis grade. The different types of infections recorded in our study are listed in Table 7.

**Table 6:** Clinical characteristics of patients who developed an infection during immunosuppressive treatment

	No. (%)		
Clinical characteristics	Infection *n* = 29	No infection *n* = 50	*P*
Cancer stage^a^			0.386
Stage III	4 (14)	12 (24)	
Stage IV	24 (86)	37 (76)	
Malignancy type			0.752
Melanoma	19 (66)	36 (72)	
Solid tumor	10 (34)	13 (26)	
Hematologic	0 (0)	1 (2)	
Immune checkpoint inhibitor type			0.758
Anti-CTLA-4	25 (86)	41 (82)	
Anti-PD-1/L1	4 (14)	9 (18)	
Treatment for diarrhea or colitis			0.483
Steroids alone	18 (62)	26 (52)	
Steroids + infliximab	11 (38)	24 (48)	
Mean length of steroid use (days, SD)	80.4 (116)	46.7 (34.5)	0.063
Length of steroid use			0.160
Short duration (≤ 30 days)	8 (28)	22 (44)	
Long duration (> 30 days)	21 (72)	28 (56)	
Diarrhea grade			0.353
Grade 1	3 (10)	10 (20)	
Grade 2–4	26 (90)	40 (80)	
Colitis grade			1.000
Grade 1	11 (38)	19 (38)	
Grade 2–3	18 (62)	31 (62)	

Abbreviations: CTLA-4, cytotoxic T-lymphocyte-associated protein-4; PD-1/L1, Programmed death-1/ligand 1; SD, standard deviation

^a^Stage of cancer was documented in 28 patients with infection and 49 patients with no infection

**Table 7:** Infections^a^ recorded in patients who received immunosuppressive treatment

Type of infection	No. (%)
Urinary tract infection	12 (38)
*Clostridium difficile* gastroenteritis	9 (28)
Pneumonia^b^	5 (16)
Disseminated *Candida* infection	2 (6)
Staphylococcal skin infection	2 (6)
Enteropathic *Escherichia coli* gastroenteritis	1 (3)
Bacteremia	1 (3)

^a^Total of 32 infections were recorded in 29 patients

^b^These 5 cases include two pseudomonas, one streptococcus, one fungal, and one viral infection

**Fig. 4:**
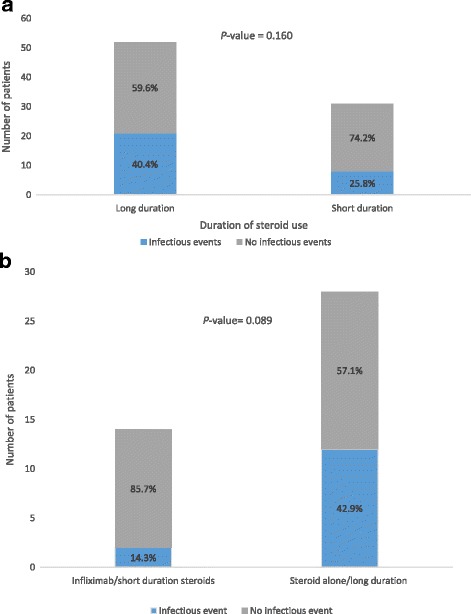
Rates of infectious events in patients given immunosuppression treatments. **a** Patients who received steroids for a long duration compared with those who received steroids for a short duration. **b** Patients who received infliximab and short-duration (≤ 30 days) steroids compared with those who received long-duration (> 30 days) steroids alone

Overall, patients who received steroids for a long duration had numerically higher rates of infection than did those who received steroids for a short duration, although the difference was not statistically significant (40.4% vs. 25.8%; Fig. 4a; *P* = 0.160). Patients who received steroids without infliximab for a long duration had a numerically higher infection rate than did those who received steroids with infliximab for a short duration, although the difference was not statistically significant (42.9% vs. 14.3%; Fig. 4b; *P* = 0.089).

We also compared the OS based on the grade of diarrhea and colitis, and a significant difference between higher grades and lower grades of toxicity was not observed (colitis grade 1 vs. grade 2/3; *P* = 0.340; diarrhea grade 1 vs. grade 2–4; *P* = 0.508, data not shown).

## Discussion

In this large-scale study, we analyzed the outcomes of 327 patients who received treatment with ICPIs, a subgroup of which developed ICPI-induced diarrhea or colitis. We found that patients with ICPI-induced diarrhea or colitis had better OS rates than those who did not develop GI symptoms (Fig. 1). Diarrhea, the most common GI-irAE, was an independent predictor of improved survival regardless of its related treatment (Fig. 1).

Prior studies have suggested a link between the development of irAEs and improved outcome [15, 19–21]. Earlier studies of ipilimumab in patients with melanoma have found an association between the development of irAEs and improved response rate and disease control [19, 20]. A study of 198 patients with melanoma or renal cell carcinoma treated with ipilimumab revealed that the 39 patients who developed enterocolitis had significantly higher tumor response rates than those who did not experience this adverse event [21]. However, another study of 298 melanoma patients treated with ipilimumab found no improved OS in those with irAEs of various types compared with no adverse events [22]. Similar conflicting results were also reported in studies of patients receiving nivolumab [15, 16]. Some of the discrepancies in results may stem from the inclusion of patients with multiple adverse events in these analyses. Also, the lack of association between pooled irAEs and survival does not rule out the possibility that individual irAEs can be associated with differences in survival [16, 22]. In our study, we included only patients with GI-irAEs, and we saw a clear difference in OS. The GI tract may be especially relevant to the study of the relationship between irAEs and response to cancer therapy. Recent studies have shown a potential association between differences in gut microbiota and differences in both survival rate and incidence of ICPI-induced colitis [23].

The median survival time for the non-diarrhea group is about 10 months, which is longer than the estimated time to GI toxicity onset (5–10 weeks) from ICPI initiation [24]. Therefore, the follow up duration in this group is adequate and will not affect our analysis result by assuming that the toxicity could have occurred if the follow-up duration was longer. Shoushtari et al [25] in their prospective study of 64 patients with melanoma treated with combination ipilimumab and nivolumab reported that 14 (56%) patients out of 25 who developed any grade diarrhea or colitis required infliximab treatment. In our study, Infliximab use was required in 35 (30%) patients who developed refractory diarrhea or colitis among the 117 patients who had diarrhea. This could be explained by the fact that our study included patients with monotherapy PD/PD-L-1 treatment not just combination therapy. Diarrhea or colitis of grade 1 may be treated conservatively without immunosuppression. However, we observed a small subset of patients who received immunosuppressant treatment for grade 1 colitis or diarrhea in our cohort. The reason behind this observation was that some patients had concurrent grade 1 diarrhea with grade 2 or above colitis and vice versa.

Looking at the baseline characteristics of patients in our cohort, we found that patients in the group with diarrhea that required immunosuppressive treatment were mostly Caucasian, and had a diagnosis of melanoma (Table 1). These findings were confirmed on the multivariate logistic regression analysis to be independent risk factors for irAE. This is in concordance with other studies showing that melanoma was much more prevalent in Caucasians, [26] and melanoma patients were found to have higher frequencies of certain irAEs, including gastrointestinal irAEs, because ipilimumab is FDA approved and most commonly used for melanoma [27]. As shown in Table 4, as well as reported in previously published studies, [6, 28] among all ICPIs, CTLA-4 agents (e.g., ipilimumab-based regimens) were associated with higher Odds Ratio for developing irAEs compared with PD-1 and PD-L1 agents (OR, 2.23; 95% CI, 1.03–4.81; *P* = 0.041). The finding of decreased risk of GI tract irAEs associated with stage IV cancer is anticipated (OR, 0.09; 95% CI, 0.03–0.30; *P* < 0.001). One of the reasons behind this observation is that patients with stage III cancer received higher dose of CTLA-4 (10 mg/kg), as approved for clinical use, compared to patients with stage IV cancer (3 mg/kg). Another reason is that PD-1 as monotherapy is approved for the treatment of patients with stage IV cancer but not stage III cancer. Furthermore, we speculate this might be also attributed to heavier tumor burdens in more advanced stage IV malignancies which may compromise the response to ICPI. It was not surprising to see that patients with irAEs stopped ICPI treatment, because of the development of toxicities, earlier than did patients without irAEs, which led to the observation of decreased risk of irAEs in patients who received longer duration of ICPI treatment.

Long-term OS is one of the most important tools that is used to evaluate the response of cancer therapy in cancer patients. The impact of irAEs due to cancer therapy on long-term OS has recently become a focus of interest for oncologists. The effects of immunosuppressive agents (corticosteroids, infliximab) that are commonly used to treat irAEs in cancer patients remain uncertain, despite the recommendation for the use of these agents in multiple practice guides [24, 29, 30]. In theory, these immunosuppressive agents may counteract the effect of ICPI, which could subsequently compromise cancer response and lead to an impaired clinical outcome. In addition, there has been a lack of consensus and good quality data on the appropriate duration of steroid use and its safety concern. Our data from a relatively large patient population demonstrated that among patients who developed ICPI-induced diarrhea or colitis, the use of immunosuppressive treatment did not affect OS (Fig. 1c), and no differences were found between patients who received steroids without infliximab and those who received steroids with infliximab (Fig. 1d). This shows that immunosuppressive treatment overall does not impact the long-term survival negatively, which is consistent with prior studies [22]. What stands out in our analysis is that diarrhea alone appeared to be a specific predictor of favorable OS (Fig. 1), regardless of the requirement for treatment. We specifically included a group of patients who had reported mild diarrhea symptoms not requiring immunosuppressive treatments in our analysis, a group that was not a focus of interest in other previously published studies. Remarkably, this additional information provided by our study improved our understanding of the role and impact of diarrhea independently on OS.

Because more patients who had diarrhea or colitis that required steroids or infliximab had stage III cancer than those who had no or mild diarrhea that did not require treatment (21% vs. 4 and 9%; Table 1), the lower cancer stage in the immunosuppressive treatment group could represent a confounding factor for improved outcomes. Therefore, we performed additional survival analysis in subgroups with different stages of cancer to further clarify this question. Interestingly, in patients with melanoma and solid tumors, no dramatic difference was observed between patients with stage IV cancer and those with stage III cancer, regardless of whether they had developed GI-irAE or not (Fig. 2). However, the small sample size of patients with stage III cancer might have underpowered our survival analysis in this subgroup.

To confirm our observation of consistently improved OS duration in patients with irAEs, after the elimination of cancer stage as a confounding factor, we performed a subgroup survival analysis of only patients with stage IV melanoma and solid tumors. A similar favorable survival pattern was found in patients with stage IV cancer who developed diarrhea or colitis, regardless of treatment requirement. Moreover, among patients who had diarrhea, no differences in OS were observed between patients who needed immunosuppressive treatment and those who did not, or between patients who received steroids without infliximab and those who received steroids with infliximab (Fig. 3), which was consistent with our results for the total cohort (Fig. 1). Therefore, our results indicate that irAEs related to ICPI, particularly GI-tract irAEs, are sensitive surrogate markers to correlate with favorable OS.

Besides long-term OS, the other outcome of interest from immunosuppressive treatment is its potential complications, such as infection. The opportunistic infections related to steroid use in our study included disseminated candida in two patients and pseudomonas pneumonia in two. We found that patients who experienced an infection received steroids for a mean duration of 80.4 days, compared with 46.7 days for those who did not have an infection (Table 6). When we arbitrarily used 30 days as the cutoff for long duration, patients who received steroids for a long duration had numerically higher rate of infection than did those who received steroids for a short duration ((40.4% vs. 25.8%;) (Fig. 4a)), but it did not reach statistical significance. In the comparison of immunosuppressive regimens, patients who received steroids for a short duration followed by infliximab had much lower infection incidence than those who received steroids alone for a long duration (Fig. 4b). This finding suggests that the early treatment with infliximab and limited steroid exposure may be beneficial.

Limitations of our study should be noted. Because there was no standard practice guideline for the management of diarrhea or colitis from oncology societies over the past 5 years, our cancer patients were mostly cared for on the basis of individual provider and institution experience and availability of resources. Even in a tertiary cancer center such as MD Anderson, our treatment of irAEs was mainly composed of long-term steroids. The use of infliximab was limited to cases that were refractory to steroids. This practice pattern certainly limited our sample size unfavorably. Because there was no clear guidance on the safe duration of steroid use for irAEs, we used an arbitrary cutoff of 30 days for our analysis, which may not be the most appropriate standard and might have affected our analysis results. Because our study population was a mixture of patients with melanoma, solid tumor, and hematologic malignancies, it would be very difficult and complicated to analyze progression-free survival given the different evaluation criteria. Instead, OS was the main outcome we measured. This may not accurately reflect cancer outcomes. Although we were able to collect the largest sample size to date from MD Anderson despite these limitations, our cohort remained underpowered for subgroup analysis.

## Conclusion

Up to this date, this study is by far the largest-scale single-center study focusing on GI tract toxicities related to ICPI use in cancer patients with long-term follow-up. Our study showed that the development of diarrhea was an independent marker and predictor of improved OS, irrespective of whether the irAE required immunosuppressive treatment or not. Treatment of diarrhea with immunosuppression did not affect OS negatively, although patients who received steroids for a long duration had a numerically higher rate of infection. These results taken together suggest that the early introduction of non-steroid immunosuppressive therapy, such as infliximab, which shorten the course of steroid treatment, may ensure an overall favorable outcome. Future prospective studies are required to further elucidate the relationship between ICPI-induced GI tract adverse events and tumor response and patient survival.

## Abbreviations

CTLA-4: Cytotoxic T-lymphocyte-associated protein-4
ECOG: Eastern Cooperative Oncology group
GI: Gastrointestinal
ICPI: Immune checkpoint inhibitor
irAE: Immune-related adverse event
OS: Overall survival
PD-1: Programmed cell death protein-1
PD-L1: Programmed death-ligand 1.
